# Microwave-Assisted Synthesis of Novel Pyrazolo[3,4-*g*][1,8]naphthyridin-5-amine with Potential Antifungal and Antitumor Activity

**DOI:** 10.3390/molecules20058499

**Published:** 2015-05-12

**Authors:** Paola Acosta, Estefanía Butassi, Braulio Insuasty, Alejandro Ortiz, Rodrigo Abonia, Susana A. Zacchino, Jairo Quiroga

**Affiliations:** 1 Heterocyclic Compounds Research Group, Department of Chemistry, Universidad del Valle, A.A. 25360 Cali, Colombia; E-Mails: paolaandrea02233@gmail.com (P.A.); braulio.insuasty@correounivalle.edu.co (B.I.); alejandro.ortiz@correounivalle.edu.co (A.O.); rodrigo.abonia@correounivalle.edu.co (R.A.); 2 Área Farmacognosia, Facultad de Ciencias Bioquímicas y Farmacéuticas, Universidad Nacional de Rosario, Suipacha 531, CP 2000 Rosario, Argentina; E-Mails: fefabutassi@hotmail.com (E.B.); szaabgil@citynet.net.ar (S.A.Z.)

**Keywords:** antifungal activity, antitumoral activity, pyrazolonaphthyridines, microwave irradiation, *Candida albicans*, *Cryptococcus neoformans*

## Abstract

The microwave assisted reaction between heterocyclic *o*-aminonitriles **1** and cyclic ketones **2** catalyzed by zinc chloride led to new series of pyrazolo[3,4-*b*][1,8]naphthyridin-5-amines **3** in good yields. This procedure provides several advantages such as being environmentally friendly, high yields, simple work-up procedure, broad scope of applicability and the protocol provides an alternative for the synthesis of pyrazolonaphthyridines. The whole series showed antifungal activities against *Candida albicans* and *Cryptococcus neoformans* standardized strains, being compounds with a 4-*p*-tolyl substituent of the naphthyridin scheleton (**3a**, **3d** and **3g**), the most active ones mainly against *C. albicans*, which appear to be related to their comparative hydrophobicity. Among them, **3d**, containing a cyclohexyl fused ring, showed the best activity. The anti-*Candida* activity was corroborated by testing the three most active compounds against clinical isolates of *albicans* and non-*albicans Candida* strains. These compounds were also screened by the US National Cancer Institute (NCI) for their ability to inhibit 60 different human tumor cell lines. Compounds **3a** and **3e** showed remarkable antitumor activity against cancer cell lines, with the most important GI_50_ values ranging from 0.62 to 2.18 μM.

## 1. Introduction

Naphthyridines are fused nitrogen heterocycles present in many natural and synthetic compounds of particular interest in Medicinal Chemistry due to their diverse biological activities. They have showed a broad range of interesting pharmacological activities, such as anti-inflammatory [1,2,3,4], analgesic, antiaggressive [5], anticancer [6], antibacterial [7], antitumor [8], antihypertensive [9] and antiallergic [10] ones. They also showed to be useful starting materials for the synthesis of various policyclic heterocycles of biological interest. Due to their biological and synthetic importance, the development of effective routes to synthesize naphthyridines continues to be an active area of research for synthetic organic chemists [11]. A survey of the literature shows that the major synthetic approaches used to prepare the naphthyridine system involved condensation of 2-aminopyridine derivatives with carbonyl compounds containing an activated methylene group [12,13,14,15,16,17,18,19] or with *β*-ketoesters [20].

Microwave irradiation (MWI) is a technique that has been employed in a number of applications in synthetic chemistry and has been observed to have several advantages compared to traditional methods of synthesis. Due to selective heating in the microwave, the occurrence of side reactions is avoided. Microwave assisted organic synthesis (MAOS) has also emerged as a powerful tool for high-throughput procedures. This can improve the yield and purity of the final compounds in short reaction times through the precise control of parameters such as power irradiation, pressure and temperature [21,22,23,24,25,26,27].

On the other hand, the Friedländer annulation is one of the most simple and straightforward approaches to the synthesis of poly-substituted pyridines and related aza-heterocycles or aza-aromatic compounds [28,29,30]. For this kind of Friedländer reaction, two points are of concern. One is the catalyst, which belongs to one of two categories: the proton acid [31,32,33,34] or the Lewis acid [35,36,37,38,39]. The other point is that the structures of a normal Friedländer condensation product are either a pyridine or a quinoline skeleton.

Cancer is the major health problem that threatens people worldwide. Since many of the current pharmacotherapeutic drugs have problems with toxicity and drug resistance, there is a strong need for the discovery and development of effective new anticancer drugs [40]. Among the wide range of compounds tested as potential anticancer agents, compounds containing the six-membered heterocyclic pyridine and/or pyrimidine, have attracted significantly attention.

In the last years, fungi have emerged as major cause of human infections especially among immunocompromised hosts having an enormous impact on morbidity and mortality [1,2,3,4]. Because most patients with invasive fungal infections are immunocompromised, the success of treatments is more dependent on the efficacy of the antifungal agent than on the immune system [41,42]. Unfortunately, the available antifungal agents are limited and it took 30 years for the newest class of antifungal drugs, the echinocandins [43,44], to appear on the market. Furthermore, the usual therapy for cryptococcal meningitis, a disease produced by *Cryptococcus neoformans* that kills most AIDs patients worldwide, is based on amphotericin B and flucytosine, which were discovered nearly 50 years ago [45]. There is, therefore, an urgent need for new antifungal chemical structures for treating infections produced by these fungi, alternatives to the existing ones [46].

Because of this great need for new antifungal and/or anticancer structures, and considering that other naphthyridines-bearing structures have shown antimicrobial and antitumor activities [4,5], herein we report the synthesis of a novel series of pyrazolo[3,4-*b*][1,8]naphthyridin-5-amines by the Friedländer condensation of *o*-aminonitrile **1** with cyclic ketones **2**. The whole series was tested for antifungal activity against standardized as well clinical strains of the clinically important fungi *C. neoformans* and species of *Candida* genus. All compounds were also evaluated to determine antitumor activity.

## 2. Results and Discussion

### 2.1. Chemistry

In our study, several conditions were tested for the first time, including diverse solvents, temperatures, heating source and catalysts in order to find the best reaction conditions for the synthesis of **3**.

**Scheme 1:**
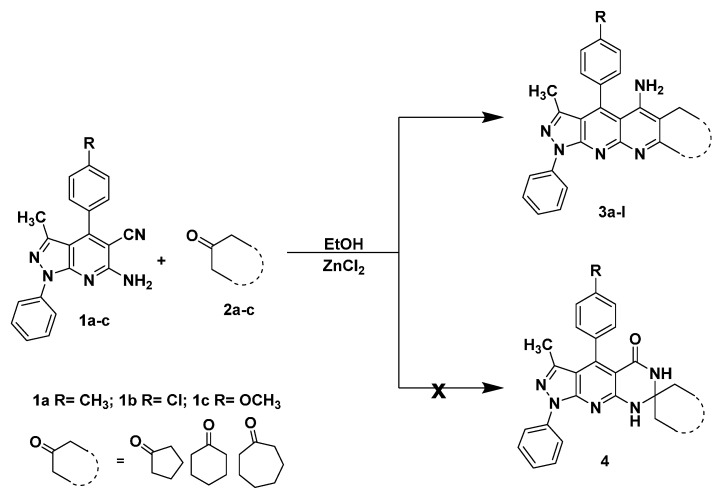
Synthesis of pyrazolo[3,4-*b*][1,8]naphthyridin-5-amine **3**.

The reactions were carried out from *o*-aminonitrile **1a** (R = CH_3_) and cyclohexanone as a model reaction (Scheme 1). When ethanol was used as the solvent and the mixture was subjected to reflux, the desired product, **3d**, was obtained in low yields (30%, 35% and 40%, entries **2**, **3** and **4**) after 10 h. In a modified protocol, the reaction was performed under MWI, obtaining significant improvements evidenced by better yields of the target product (40%, 45% and 75%, entries **6**, **7** and **8**) and shorter reaction times. As it is clearly observed in entries **1** and **5**, the reaction did not take place without a catalyst. So, we performed the reaction with a Lewis acid catalyst. Results showed that both the aluminum chloride and the *p*-toluenesulfonic acid, in either conventional refluxing or MWI, favored the product of Friedländer condensation **3** (Table 1), but the yields were not very high, whereas anhydrous zinc chloride favored the formation of the product in higher yields in both conditions. The best catalyst, anhydrous zinc chloride, was therefore chosen as the catalyst for this new transformation.

**Table 1:** Optimization of reaction conditions for the synthesis of compounds **3d**.

Entries	Catalyst	Conditions	Yield (%)
1	–	EtOH, reflux	–
2	AlCl_3_	EtOH, reflux	30
3	*p*MeC_6_H_4_SO_3_H	EtOH, reflux	35
4	ZnCl_2_	EtOH, reflux	40
5	–	EtOH, MW (120 °C, 300 W)	–
6	AlCl_3_	EtOH, MW (120 °C, 300 W)	40
7	*p*MeC_6_H_4_SO_3_H	EtOH, MW (120 °C, 300 W)	45
8	ZnCl_2_	EtOH, MW (120 °C, 300 W)	75

To determine the extent of application of the cyclization reaction, the same conditions were used for some selected cyclic ketones (of five, six and seven members) to get the pyrazolo[3,4-*b*][1,8]naphthyridin-5-amines (Scheme 1, Table 2). 

**Table 2:** Scope of the reaction.

Compound 3	Structure	Yield (%)
**3a**	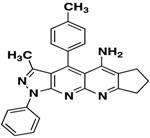	**65**
**3b**	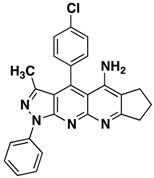	**63**
**3c**	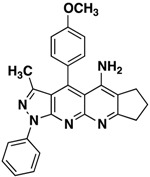	**62**
**3d**	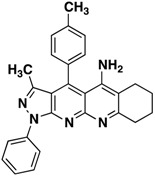	**80**
**3e**	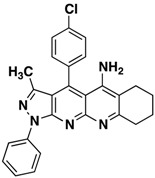	**75**
**3f**	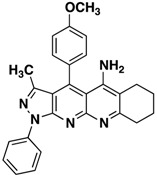	**65**
**3g**	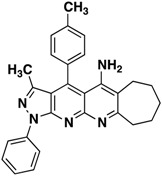	**60**
**3h**	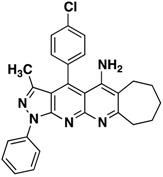	**60**
**3i**	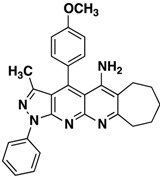	**55**

All the structures **3a**–**i** were characterized by IR (Infrared), ^1^H-NMR, ^13^C-NMR (Proton- and carbon-Nuclear Magnetic Resonance), MS (mass-spectrometry) spectra and elemental analyses. Formation of the pyrazolo[3,4-*b*][1,8]naphthyridin-5-amines **3** was unequivocally established by NMR data of the products. The chemical shifts and multiplicities of the protons were in accordance with the expected values. For example, signals for the protons of the phenyl of compounds **3** were found between 7.20 and 8.30 ppm. The signal for NH_2_ appears as a broad singlet between 6.10 and 6.80 ppm, the signals of methyl protons of CH_3_ appears as singlets between 1.74 and 1.84 ppm and all aliphatic protons, corresponding to the five-, six- and seven-membered, appear between 1.46 and 3.87.

**Scheme 2:**
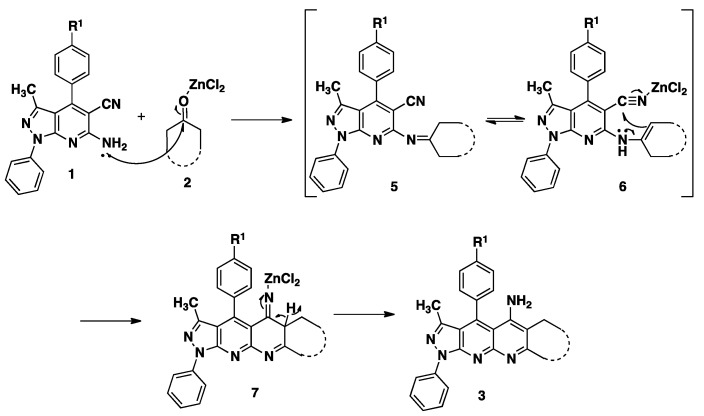
Proposed mechanism.

A possible mechanism of the cyclization reaction between **1** and **2** is depicted in Scheme 2. The reaction seemed to proceed via the initial formation of the imine intermediate **5** by the normal Friedländer reaction [47,48], which subsequently affords the final compound **3** by an intramolecular nucleophilic cyclization on the nitrile.

### 2.2. Antifungal Activity

The antifungal properties of compounds **3a**–**3i** were tested first against two clinically important fungal species, *Candida albicans* and *C. neoformans*, which were selected due to the following facts: *C. albicans* is among the most common cause of opportunistic fungal infections in immunocompromised hosts, although recently, non-*albicans Candida* species have been increasingly identified among *Candida*-infected patients [49].

In turn, *C. neoformans* is the most frequent cause of meningitis and is one of the most important HIV-related fatal opportunistic mycosis, which has killed more than 650,000 immunocompromised patients worldwide up to date [46]. Although the incidence of disease tends to decline in countries with highly active anti-retroviral therapy, the outcome of infection is influenced by a variety of factors including the antifungal resistance and new strategies including new structural types with anti-cryptococcal activity are highly welcome [50].

For a more comprehensive analysis of the antifungal results, we grouped the compounds in two series, (i) and (ii). Series (i) includes compounds with different rings (cyclopentyl, cyclohexyl or cycloheptyl) fused to the naphthyridines scheleton and the same R (sub-series i.1 with CH_3_, i.2 with Cl or i.3 with OCH_3_), which allowed having a look at the influence of the size of the fused ring on the antifungal activity. Series (ii) includes compounds with different R (CH_3_, Cl or OCH_3_), but the same fused ring moiety (subseries ii.1 with cyclopentyl; ii.2 with cyclohexyl and ii.3 with cycloheptyl), which allowed analyzing the role played by the different R substituents in the antifungal activity.

Compounds were evaluated by using the standardized microbroth dilution method M-27A3 for yeasts of Clinical and Laboratory Standards Institute [51], which assures confident and reproducible results.

Results of the whole series are expressed as the percentages of inhibition of each fungus in the range 250–3.9 µg∙mL^−1^ and are presented as Supplementary Table 1 (Table S1).

For the sake of clarity, Table S1 was summarized in Table 3 by using the MICs at different endpoints, such as MIC_100_ MIC_80_ and MIC_50_ (minimum concentration that inhibits 100%, 80% and 50% of growth) that have showed to consistently represent the *in vitro* activity of compounds [52].

From Table 3, it is clear that all compounds displayed some degree of activity against *C. albicans* and *C. neoformans*. However, *C. albicans* showed to be more sensitive for the whole series than *C. neoformans*, since eight of the nine compounds showed MIC_100_ against *C. albicans* below 250 µg∙mL^−1^ (range = 31.2–250 µg∙mL^−1^), while only four of the nine compounds showed MICs_100_ below 250 µg∙mL^−1^ against *C. neoformans* (range = 125–250 µg∙mL^−1^). The same analysis can be performed with MIC_80_ and MIC_50_.

Regarding the activity against *C. albicans*, the comparison of the activity of compounds with same ring and different R (compare **3a**/**3b**/**3c**, **3d/3e/3f** or **3g/3h/3i**) showed that the type of ring does not play a crucial role in the activity, since the most active compounds, **3a**, **3d** and **3g**, possess different rings, cyclopentyl, cyclohexyl and cycloheptyl, respectively. Instead, the three compounds share the feature of having a 4-*p*-tolyl moiety.

**Figure 1:**
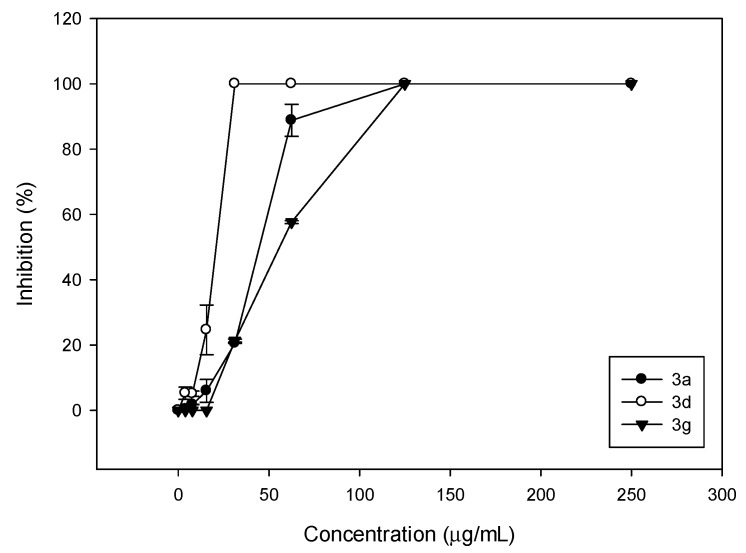
Comparative curves of the growth inhibition of *C. albicans* ATCC 10231 produced by compounds **3a**, **3d** and **3g** at different concentrations. Inhibition percentages are the means ± SD obtained from experiments in triplicate.

The comparison of the activity of the three compounds possessing a methyl groups as R can be clearly observed in Figure 1.

From Figure 1, it is clear that **3d**, possessing a cyclohexyl ring, is the most active compound, while **3a** and **3g** (with cyclopentyl and cycloheptyl, respectively) possessed lower activities than **3d**, but similar to each other.

**Table 3:** Minimum inhibitory concentrations (MIC_100_, MIC_80_ and MIC_50_) and minimum fungicidal concentrations (MFC) of **3a**–**i** grouped by their structural features against standardized strains of *Candida albicans* and *C. neoformans*. 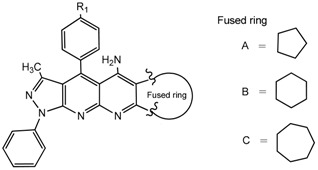

R1	Fused Ring	Comp	*C. albicans* ATCC 10231			*C. neoformans* ATCC 32264		
			MIC_100_	MIC_80_	MIC_50_	MIC_100_	MIC_80_	MIC_50_
	A	**3a**	125	62.5	62.5	250	250	7.8
CH_3_	B	**3d**	31.2	31.2	31.2	250	125	125
	C	**3g**	125	62.5	62.5	125	125	125
	A	**3b**	>250	250	125	>250	250	250
Cl	B	**3e**	250	125	125	>250	>250	250
	C	**3h**	125	125	125	250	125	125
	A	**3c**	250	250	250	>250	250	31.2
OCH_3_	B	**3f**	250	125	125	250	125	125
	C	**3i**	250	125	125	>250	>250	250
Amph B			0.12			0.25		

**Figure 2:**
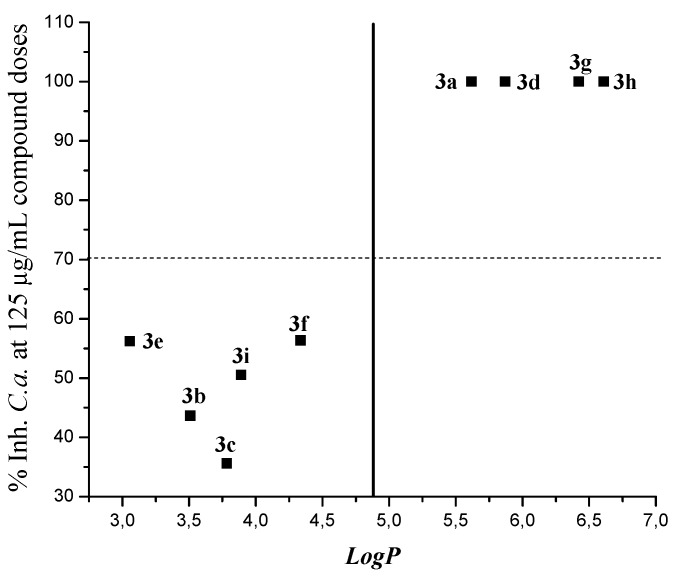
Log*P vs.* inhibition percentage of *C. albicans* growth, by **3a**–**i** at 125 µg∙mL^−1^.

It is known that log*P* (the logarithm of the partition coefficient in a biphasic system, e.g., *n*-octanol/water) describes the macroscopic hydrophobicity of a molecule, which is a factor that determines its ability to penetrate fungal cell membranes and to reach the interacting sites, thus influencing the antifungal activity of compounds [53,54]. In order to establish a correlation between log*P* and the activity of 3a–i, if any, log*P* of each compound was calculated and correlated to the percentage of inhibition of each compound at a selected concentration (125 µg∙mL^−1^). Table 4 shows the values of log*P* and Figure 2 plots log*P vs.* activity of all compounds of the series. For the calculation of log*P*, we used quantum mechanical at semi-empirical level using Mopac, with the parametric method 3 (PM3). The molecular modeling were prepared using CS Chem-Office Software version 9.0 (Cambridge software) [55]. The models were minimizedation until the root mean square (RMS) gradient value reached a value smaller than 0.0001 kcal∙mol^−1^. The lowest energy structure was used for each molecule to calculate log*P* values.

**Table 4:** *In vitro* activity of compounds **3a**–**i** expressed as % inhibition of *Candida albicans* (*C.a*.) ATCC 10231 growth at 125 µg∙mL^−1^.

Compound	Log *P*	% Ihn *C.a.*
**3a**	5.61803	100
**3b**	3.50884	43.63
**3c**	3.78334	35.58
**3d**	5.87099	100
**3e**	3.05581	56.19
**3f**	4.33631	56.35
**3g**	6.42396	100
**3h**	6.60877	100
**3i**	3.88927	50.55

Interesting enough, the most active compounds, **3a**, **3d**, **3g** and **3h**, possess log*P* values between 5.62 and 6.61. The rest of these compounds possess values of log*P* lower than 4.8 (mean value). The results showed above suggest that the antifungal activity of compounds **3** would be related to their hydrophobicity.

#### Second-Order Studies with Clinical Isolates

In order to gain insight into the actual inhibitory capacity of **3a**, **3d** and **3g** against *C. albicans*, the three compounds were tested not only against the ATCC standardized strain but also on six clinical strains of *C. albicans* (isolated from patients suffering from mycoses) and on four non-*albicans Candida* strains, such as *C. glabrata*, *C. parapsilopsis*, *C. krusei* and *C. tropicalis*, all of them provided by CEREMIC (see Experimental). The selection of these non-*Candida* spp. was due to that fact that these four spp, along with *C. albicans*, are responsible for more than 90% of all *Candida* infection all over the world and also in Latin American countries [49].

The minimum inhibitory concentration (MIC) values of **3a**, **3d** and **3g** were determined against this new panel by determining MIC_100_, MIC_80_ and MIC_50_. These results are shown in Table 5.

**Table 5:** The 100%, 80% and 50% inhibitory concentrations (MIC_100_, MIC_80_ and MIC_50_) of **3a**, **3d**, and **3g** against clinical isolates of *C. albicans* and *non-albicans Candida* strains. For the sake of comparison, MIC_100_, MIC_80_ and MIC_50_ of all compounds against the ATCC 10231 standardized strain of *C. albicans* are included.

Strain	Voucher Specimen	3a			3d			3g			Amph.
		MIC_100_	MIC_80_	MIC_50_	MIC_100_	MIC_80_	MIC_50_	MIC_100_	MIC_80_	MIC_50_	MIC_100_
*C. albicans*	ATCC 10231	125	62.5	62.5	31.2	31.2	31.2	125	62.5	62.5	1.00
*C. albicans*	CCC 125	62.5	62.5	62.5	31.2	31.2	31.2	125	62.5	31.2	0.78
*C. albicans*	CCC 126	125	62.5	62.5	31.2	31.2	15.6	125	62.5	62.5	1.56
*C. albicans*	CCC 127	62.5	31.2	31.2	31.2	31.2	31.2	125	62.5	31.2	0.78
*C. albicans*	CCC 128	125	62.5	31.2	31.2	31.2	15.6	125	62.5	62.5	1.56
*C. albicans*	CCC 129	125	31.2	62.5	62.5	31.2	31.2	250	62.5	31.2	0.78
*C. albicans*	CCC 130	125	62.5	62.5	31.2	31.2	15.6	125	31.2	31.2	0.50
*C. glabrata*	CCC 115	>250	>250	>250	>250	>250	>250	>250	>250	250	0.39
*C. parapsilopsis*	CCC 124	125	62.5	62.5	31.2	31.2	31.2	125	125	62.5	0.78
*C. krusei*	CCC 117	125	125	62.5	15.6	15.6	7.8	125	62.5	62.5	0.39
*C. tropicalis*	CCC 131	125	125	62.5	31.2	15.6	15.6	125	125	62.5	0.50

ATCC = American Type Culture Collection (Illinois, USA); CCC = Center of Mycological Reference (Rosario, Argentina), *C. albicans* = *Candida albicans*; *C. glabrata = Candida glabrata*; *C. parapsilopsis = Candida parapsilopsis*; *C. krusei = Candida krusei*; *C. tropicalis = Candida tropicalis*; Amph. = Amphotericin B.

As it can be seen in Table 5, clinical isolates of *Candida* genus showed similar sensitivity to **3a**, **3d** and **3g** than the standardized strain *C. albicans* ATCC 10231, corroborating the anti-*Candida* activity of the three compounds.

### 2.3. Anticancer Activity

As a preliminary screening, structures of all new compounds (*i.e.*, **3a**–**j**) were submitted to the Developmental Therapeutics Program (DTP) at National Cancer Institute (NCI) for evaluation of their anticancer activity against different human cell lines. All the submitted structures (**3a**–**j**) were selected and subjected to the preliminary evaluation against the 60 tumor cell lines at a single dose of 10 mM after 48 h of incubation. The output from the single dose screening was reported as a mean graph available for analysis by the COMPARE program (data not shown). The results of this first assay showed that compounds **3a** and **3e** were active.

Then, the second screening was made in order to determine cytostatic activity of active compound against the 60 tumor cell lines represented in leukemia, melanoma, lung, colon, brain, breast, ovary, kidney and prostate panels; where the testing results were expressed according to the following three parameters: GI_50_, which is the molar concentration of the compounds required to inhibit the growing of the cell lines to 50% (relative to untreated cells). TGI as the molar concentration that causes total growth inhibition, and LC_50_, which is a parameter of cytotoxicity and reflects the molar concentration needed to kill 50% of the cells [56]. The active compounds were evaluated at five concentration levels (100, 10, 1.0, 0.1, and 0.01 mM) and the test consisted of a 48 h continuous drug exposure protocol using sulforhodamine B (SRB) protein assay to estimate cell growth. Details of this evaluation method, and the complementary information related with the activity pattern over all cell lines, have been published [57,58,59,60,61]. The compound **3a** show a remarkable activity against 39 human tumor cell lines (Table 6), with low values of GI_50_ ≈ 10^−6^ μM, being SR (Leukemia; GI_50_ = 0.62 μM, LC_50_ > 100 μM), HCT-15 (Colon Cancer; GI_50_ = 1.98 μM, LC_50_ > 100 μM) and MCF7 (Breast Cancer; GI_50_ = 1.62 μM, LC_50_ > 100 μM) the most sensitive strains. In a similar way, compound **3e** also showed an interesting activity against 41 human tumor cell lines, remarking that for SR and CCRF-CEM (Leukemia) with GI_50_ Values of 1.84, 2.18 μM and LC_50_ values of 79.5 μM and >100 μM, respectively. The cytotoxic effects associated with compounds **3a** and **3e** were measured as LC_50_ goes from 6.79 to >100 μM, indicating a low toxicity of these compounds for normal human cell lines, as required for development of potential antitumor agents.

**Table 6:** *In vitro* testing expressed as growth inhibition of cancer cell lines for compounds **3a** and **3e** ^a^.

Panel/Cell Line	Compounds			
	3a		3e	
	GI50 ^b^ (µM)	LC50 ^c^ (µM)	GI50 ^b^ (µM)	LC50 ^c^ (µM)
*Leukemia*				
CCRF-CEM	2.56	>100	2.18	>100
HL-60(TB)	2.24	>100	2.12	8.71
K-562	2.78	>100	2.30	49.1
MOLT-4	3.17	>100	2.30	42.4
RPMI-8226	2.25	>100	1.92	9.72
SR	0.62	>100	1.84	79.5
*Non-small Cell Lung Cancer*				
A549/ATCC	2.22	40.2	3.01	46.7
HOP-62	3.32	58.7	14.1	60.9
HOP-92	–	–	–	–
NCI-H226	21.6	>100	19.6	>100
NCI-H23	16.3	94.0	–	–
NCI-H322M	3.26	33.2	2.92	33.1
NCI-H460	1.92	8.64	3.28	47.5
NCI-H522	20.1	93.0	2.91	56.0
*Colon Cancer*				
COLO 205	1.74	7.90	5.91	31.3
HCC-2998	–	–	3.51	53.8
HCT-116	1.68	–	2.15	25.9
HCT-15	1.98	>100	3.08	55.7
HT29	14.3	73.2	2.68	41.9
KM12	4.83	50.4	3.16	40.0
SW-620	2.48	48.9	3.05	50.3
*CNS Cancer*				
SF-268	3.13	72.8	5.33	54.6
SF-295	2.99	58.3	10.1	54.0
SF-539	1.88	6.49	14.5	58.8
SNB-19	3.46	90.6	10.8	66.4
SNB-75	2.43	46.5	14.0	72.1
U251	1.78	6.85	1.72	7.36
*Melanoma*				
LOX IMVI	1.88	6.79	12.0	60.8
MALME-3M	21.5	74.5	–	–
M14	3.50	59.0	3.74	46.6
MDA-MB-435	17.1	67.3	3.83	53.4
SK-MEL-2	21.4	89.2	2.63	35.3
SK-MEL-28	11.3	62.6	6.75	70.2
SK-MEL-5	1.77	67.3	1.84	8.08
UACC-257	14.3	61.6	2.43	33.3
UACC-62	–	–	13.7	56.8
*Ovarian Cancer*				
IGROV1	4.43	95.3	4.26	81.5
OVCAR-3	2.06	9.47	4.17	43.3
OVCAR-4	3.95	>100	3.22	96.0
OVCAR-5	15.6	72.4	10.1	53.2
OVCAR-8	2.32	>100	2.82	48.0
NCI/ADR-RES	15.0	>100	3.41	>100
SK-OV-3	19.9	61.8	12.1	50.3
*Renal Cancer*				
786-0	2.03	–	4.55	50.4
A498	16.2	64.1	11.9	62.7
ACHN	1.94	7.58	4.32	46.3
CAKI-1	2.56	31.5	3.88	43.4
RXF 393	–	–	8.48	76.0
SN12C	1.84	7.40	3.72	55.9
TK-10	–	–	–	–
UO-31	1.84	7.33	2.46	41.3
*Prostate Cancer*				
PC-3	7.05	67.3	2.54	42.0
DU-145	2.40	17.5	4.72	45.6
*Breast Cancer*				
MCF7	1.62	>100	2.57	74.0
MDA-MB-231/ATCC	1.93	8.47	4.15	47.5
HS 578T	12.1	>100	12.5	100
BT-549	24.4	>100	17.2	66.5
T-47D	2.21	>100	–	–
MDA-MB-468	2.03	30.2	2.33	40.5

^a^ Data obtained from NCI’s *in vitro* disease-oriented human tumor cell lines screen [57]; ^b^ GI_50_ was the drug concentration resulting in a 50% reduction in the net protein increase (as measured by SRB staining) in control cells during the drug incubation. Determined at five concentration levels (100, 10, 1.0, 0.1, and 0.01 μM); ^c^ LC_50_ is a parameter of cytotoxicity and reflects the molar concentration needed to kill 50% of the cells.

## 3. Experimental Section

### 3.1. General Information

All the reagents were commercially available and used without any further purification. The solvents used were of analytical grade. Microwave experiments were carried out on a focused microwave reactor (300W CEM Discover). TLC analyses were performed on Merck TLC-plates aluminum silica gel 60 F_254_. Melting points were determined in a Büchi Melting Point Apparatus and are uncorrected. The IR analysis was performed on a Shimadzu FTIR 8400 spectrophotometer in KBr disks. ^1^H- and ^13^C-NMR spectra were run on a Bruker AVANCE 400 spectrometer operating at 400 MHz and 100 MHz, respectively, using dimethylsulfoxide-*d*_6_ as solvents and tetramethylsilane as internal standard. The mass spectra were scanned on a Shimadzu GCMS-QP 2010 and Hewlett-Packard HP Engine-5989 spectrometer (equipped with a direct inlet probe) and operating at 70 eV. The elemental analyses were obtained using a LECO CHNS-900 elemental analyzer and the values are within ± 0.4% of the theoretical values.

### 3.2. Chemistry

#### General Procedure for the Synthesis of Pyrazolo[4,3-*g*][1,8]naphthyridin-5-amine

All experiments were carried out using a focused microwave reactor (CEM Discover TM). A mixture of *ortho*-aminonitrile **1** **(** 0.3 mmol), an excess of cyclic ketone **2** and ZnCl_2_ (10 mol %), were subjected to microwave irradiation, at 120 °C for 5–10 min and a maximum power of 300 W. Then, the solvent in the reaction mixture was removed under reduced pressure. Purification of products was performed using column chromatography in a mixture CHCl_3_/EtOH (20:1) as eluent.

*3-Methyl-1-phenyl-4-(p-tolyl)-6,7-dihydro-8H-cyclopenta[g]pyrazolo[3,4-b][1,8]naphthyridin-5amine* (**3a**). Yellow solid, yield 65%, mp 224–225. FTIR (KBr) ν(cm^−1^): 3446 (NH), 3030 (=C-H), 1620, 1569 (C=N and C=C). ^1^H-NMR (400 MHz, DMSO-*d*_6_): 1.86 (s, 3H), 2.21 (t, 2H, *J* = 7.2 Hz), 2.77 (s, 3H), 2.75 (t, 2H, *J* = 7.3 Hz), 3.22 (t, 2H, *J* = 7.6 Hz), 7.39 (t, 1H, *J* = 7.4 Hz), 7.56–7.64 (m, 6H), 8.30 (d, 2H, *J* = 7.8 Hz). Not observed (brs, 2H, NH_2_). ^13^C-NMR (100 MHz DMSO-*d*_6_) δ: 14.0 (CH_3_), 21.5 (CH_3_), 22.1 (CH_2_), 28.2 (CH_2_), 32.9 (CH_2_), 104.7 (C), 114.7 (C), 116.6 (C), 120.3 (CH), 126.2 (C), 128.0 (CH), 129.3 (CH), 130.2 (CH), 130.8 (CH), 138.2 (C), 140.4 (C), 145.3 (C), 147.1 (C), 148.1 (C), 149.2 (C), 156.5 (C), 161.1 (C). HR-MS calcd for C_26_H_23_N_5_ 405, 1953, found [M+K]^+^ 443.9281. [M+H]^+^ 405.8378.

*4-(4-Chlorophenyl)-3-methyl-1-phenyl-6,7-dihydro-8H-cyclopenta[g]pyrazolo[3,4b][1,8]naphthyridin-5-amine* (**3b**). Yellow solid, yield 63%, mp 206–207. FTIR (KBr) ν(cm^−1^): 3467 (NH), 3035 (=C-H), 1622, 1568 (C=N and C=C).^1^H-NMR (400 MHz, DMSO-*d*_6_): 1.84 (s, 3H), 2.14–224 (m, 2H), 2.70–2.78 (m, 2H), 3.17–3.22 (m, 2H), 6.61 (brs, 2H, NH_2_), 7.37 (t, 1H, *J* = 7.4 Hz), 7.59 (t, 2H, *J* = 7.7 Hz), 7.73 (d, 2H, *J* = 8.0 Hz), 7.84 (d, 2H, *J* = 8.4 Hz), 8.30 (d, 2H, *J* = 6.7 Hz). ^13^C-NMR (100 MHz DMSO-*d*_6_) δ: 14.2 (CH_3_), 21.7 (CH_2_), 27.9 (CH_2_), 32.9 (CH_2_), 104.9 (C), 115.0 (C), 118.1 (C), 120.3 (CH), 126.1 (CH), 129.3 (CH), 129.8 (CH), 130.4 (CH), 132.9 (C), 135.5 (C), 138.3 (C), 144.9 (C), 145.4 (C), 147.5 (C), 149.1 (C), 156.4 (C), 160.1 (C). EI MS (70 eV): *m/z*: 425/427(M^+^, 1/0.35), 410/412(3/1), 236 (17). Anal. Calcd for C_25_H_20_ClN_5_: C, 70.50; H, 4.73; N, 16.44; found: C, 70.55; H, 4.68; N, 16.49.

*4-(4-Methoxyphenyl)-3-methyl-1-phenyl-6,7dihydro-8H-cyclopenta[b]pyrazolo[3,4-g][1,8]naphthyridin-5-amine* (**3c**). Yellow solid, yield 62%, mp 164–165. FTIR (KBr) ν(cm^−1^): 3450 (NH), 3035 (=C-H), 1625, 1565 (C=N and C=C). ^1^H-NMR (400 MHz, DMSO-*d*_6_): 1.78 (s, 3H), 2.09–2.20 (m, 2H), 2.67 (s, 2H), 3.19 (s, 2H,), 3.87 (s, 3H), 7.25 (d, 2H, *J* = 8.6 Hz), 7.31 (t, 1H, *J* = 7.1 Hz), 7.51 (s, 4H), 8.21 (d, 2H, *J* = 8.6 Hz). Not observed (brs, 2H, NH_2_). ^13^C-NMR (100 MHz DMSO-*d*_6_) δ: 14.4 (CH_3_), 21.8 (CH_2_), 28.1 (CH_2_), 32.8 (CH_2_), 56.0 (CH_3_), 105.4 (C), 109.8 (C), 115.3 (CH), 120.4 (C), 120.8 (CH), 125.4 (C), 126.1 (C), 126.4 (CH), 129.6 (CH), 130.0 (CH), 136.3 (C), 145.4 (C), 146.6 (C), 148.2 (C), 149.4 (C), 155.0 (C), 161.0 (C). *m/z*: 421 (M^+^, 1), 406 (3), 236 (10). Anal. Calcd for C_26_H_23_N_5_O: C, 74.09; H, 5.50; N, 16.62; found: C, 74.05; H, 5.56; N, 16.59.

*3-Methyl-1-phenyl-4-(p-tolyl)-6,7,8,9-tetrahydro-1H-benzo[g]pyrazolo[3,4-b][1,8]naphthyridin-5-amine* (**3d**). Yellow solid, yield 80%, mp 161-162. FTIR (KBr) ν(cm^−1^): 3444 (NH_2_), 2953 (=C-H), 1626, 1589 (C=N and C=C). ^1^H-NMR (400 MHz, DMSO-*d*_6_): 1.82 (s, 7H), 2.34 (s, 2H), 2.51 (s, 3H), 2.98 (s, 2H), 6.71 (brs, 2H, NH_2_), 7.36 (t, 1H, *J* = 7.4 Hz), 7.53–7.63 (m, 6H), 8.31 (d, 2H, *J* = 7.8 Hz). ^13^C-NMR (100 MHz DMSO-*d*_6_) δ: 14.5 (CH_3_), 21.2 (CH_2_), 21.5 (CH_3_), 21.6 (CH_2_), 23.0 (CH_2_) 29.7 (CH_2_), 104.8 (C), 109.3 (C), 116.9 (C), 120.5 (CH), 126.3 (C), 128.5 (CH), 129.7 (CH), 130.7 (CH), 131.7 (CH), 138.9 (C), 140.7 (C), 145.6 (C), 147.1 (C), 149.2 (C), 149.8 (C), 156.6 (C), 156.9 (C). HR-MS calcd for C_27_H_25_N_5_ 419.2110, found. [M+H]^+^ 420.005.

*4-(4-Chlorophenyl)-3-methyl-1-phenyl-6,7,8,9-tetrahydro-1H-benzo[g]pyrazolo[3,4-b][1,8]naphthyridin-5-amine* (**3e**). Yellow solid, yield 75%, mp 180–181. FTIR (KBr) ν(cm^−1^): 3448 (NH_2_), 2960 (=C-H), 1628, 1579 (C=N and C=C).^1^H-NMR (400 MHz, DMSO-*d*_6_): 1.84 (s, 4H), 1.88 (s, 3H), 2.38 (s, 2H), 3.02 (s, 2H), 7.40 (t, 1H, *J* = 7.2 Hz), 7.62 (t, 2H, *J* = 7.4 Hz), 7.75 (d, 2H, *J* = 8.4 Hz), 7.87 (d, 2H, *J* = 8.4 Hz), 8.29 (d, 2H, *J* = 8.0 Hz). Not observed (brs, 2H, NH_2_). ^13^C-NMR (100 MHz DMSO-*d*_6_) δ: 14.3 (CH_3_), 20.4 (CH_2_), 20.9 (CH_2_), 22.4 (CH_2_) 28.1 (CH_2_), 104.0 (C), 109.1 (C), 116.9 (C), 120.4 (CH), 126.3 (CH), 129.4 (CH), 129.9 (CH), 130.3 (CH), 132.5 (C), 135.7 (C), 135.9 (C), 138.2 (C), 145.3 (C), 145.7 (C), 149.4 (C), 153.6 (C), 157.6 (C). *m/z*: 439/441 (M^+^,1/0.35), 424/426 (4/1.3), 236 (20). Anal. Calcd for C_26_H_22_ClN_5_: C, 70.98; H, 5.08; N, 15.92; found: C, 70.94; H, 5.12; N, 15.88.

*4-(4-Methoxyphenyl)-3-methyl-1-phenyl-6,7,8,9-tetrahydro-1H-benzo[g]pyrazolo[3,4-b][1,8]naphthyridin-5-amine* (**3f**). Yellow solid, yield 65%, mp 135–136. FTIR (KBr) ν(cm^−1^): 3450 (NH_2_), 2965 (=C-H), 1617, 1520 (C=N and C=C). ^1^H-NMR (400 MHz, DMSO-*d*_6_): 1.84 (s, 4H), 1.88 (s, 3H), 2.38 (s, 2H), 3.02 (s, 2H), 3.93 (s, 3H), 7.40 (t, 1H, *J* = 7.2 Hz), 7.62 (t, 2H, *J* = 7.4 Hz), 7.75 (d, 2H, *J* = 8.4 Hz), 7.87 (d, 2H, *J* = 8.4 Hz), 8.29 (d, 2H, *J* = 8.0 Hz). Not observed (brs, 2H, NH_2_). ^13^C-NMR (100 MHz DMSO-*d*_6_) δ: 14.2 (CH_3_), 20.3 (CH_2_), 20.9 (CH_2_), 22.3 (CH_2_) 27.9 (CH_2_), 55.6 (CH_3_), 104.2 (C), 108.9 (C), 115.3 (CH), 117.3 (CH), 120.3 (CH), 125.3 (C), 126.2 (C), 129.3 (CH), 129.7 (CH), 138.3 (C), 145.6 (C), 147.2 (C), 147.3 (C), 149.3 (C), 154.2 (C), 158.1 (C), 160.8 (C). HR-MS calcd for C_27_H_25_N_5_O 435.2059, found [M+H]^+^ 436.1918.

*3-Methyl-1-phenyl-4-(p-tolyl)-6,7,8,9-tetrahydro-10H-cyclohepta[g]pyrazolo[3,4-b][1,8]naphthyridin-5-amine* (**3g**). Yellow solid, yield 60%, mp 177–178. FTIR (KBr) ν(cm^−1^): 3489 (NH_2_), 3.040 (=C-H), 1630, 1569 (C=N and C=C). ^1^H-NMR (400 MHz, DMSO-*d*_6_): 1.46 (s, 2H), 1.68 (s, 2H), 1.74 (s, 3H), 1.77 (s, 2H), 2.46 (s, 3H), 2.63 (s, 2H), 3.11 (s, 2H), 7.33 (t, 1H, *J* = 7.4 Hz), 7.60–7.46 (m, 6H), 8.20 (d, 2H, *J* = 7.9 Hz). Not observed (brs, 2H, NH_2_). ^13^C-NMR (100 MHz DMSO-*d*_6_) δ: 14.7 (CH_3_), 21.8 (CH_3_), 24.9 (CH_2_), 25.8 (CH_2_), 26.3 (CH_2_), 31.3 (CH_2_), 33.8 (CH_2_), 100.3 (C), 105.3 (C), 114.9 (C), 117.8 (C), 121.0 (CH), 127.0 (CH), 128.7 (CH), 130.0 (CH), 131.0 (CH), 131.3 (C), 138.8 (C), 141.2 (C), 144.4 (C), 146.1 (C), 147.8 (C), 149.6 (C), 157.4 (C). *m/z*: 433 (M^+^,1), 424 (4/1.3), 236 (25). Anal. Calcd for C_28_H_27_N_5_: C, 77.57; H, 6.28; N, 15.16; found: C, 77.52; H, 6.33; N, 15.11.

*4-(4-Chlorophenyl)-3-methyl-1-phenyl-6,7,8,9-tetrahydro-10H-cyclohepta[g]pyrazolo[3,4-b][1,8]naphthyridin-5-amine* (**3h**). Yellow solid, yield 60%, mp 146–147. FTIR (KBr) ν(cm^−1^): 3480 (NH_2_), 3035 (=C-H), 1620, 1518 (C=N and C=C). ^1^H-NMR (400 MHz, DMSO-*d*_6_): 1.51–1.59 (m, 2H), 1.71–1.79 (m, 2H), 1.81–1.87 (m, 2H), 1.88 (s, 3H), 2.72–2.77 (m, 2H), 3.17–3.23 (m, 2H), 7.41 (t, 1H, *J* = 7.4 Hz), 7.63 (t, 2H, *J* = 8.0 Hz), 7.76 (d, 2H, *J* = 8.4 Hz), 7.86 (d, 2H, *J* = 8.4 Hz). 8.32 (d, 2H, *J* = 7.8 Hz). Not observed (brs, 2H, NH_2_). ^13^C-NMR (100 MHz DMSO-*d*_6_) δ: 14.4 (CH_3_), 24.3 (CH_2_), 25.3 (CH_2_), 25.9 (CH_2_), 30.8 (CH_2_), 42.6 (CH_2_), 104.2 (C), 109.3 (C), 117.1 (C), 120.5 (CH), 126.4 (CH), 129.5 (CH), 129.8 (CH), 130.5 (CH) 132.3 (C), 135.4 (C), 135.6 (C), 138.7 (C), 145.8 (C), 145.4 (C), 149.7 (C), 153.8 (C), 157.3 (C). *m/z*: 453/455 (M^+^,3/1), 438 (1/0.33), 342 (15), 236 (30). Anal. Calcd for C_27_H_24_ClN_5_: C, 71.43; H, 5.33; N, 15.43; found: C, 71.47; H, 5.28; N, 15.47.

*4-(4-Methoxyphenyl)-3-methyl-1-phenyl-6,7,8,9-tetrahydro-10H-cyclohepta[g]pyrazolo[3,4-b][1,8]naphthyridin-5-amine* (**3i**). Yellow solid, yield 55%, mp 125–126. FTIR (KBr) ν(cm^−1^): 3489 (NH_2_), 3038 (=C-H), 1618, 1525 (C=N and C=C). ^1^H-NMR (400 MHz, DMSO-*d*_6_): 1.48–1.53 (m, 2H), 1.68–1.75 (m, 2H), 1.77–1.85 (m, 5H), 2.66–2.72 (m, 2H), 3.13–3.20 (m, 2H), 3.89 (s, 3H), 7.30 (d, 2H, *J* = 8.6 Hz), 7.46 (t, 1H, *J* = 7.4 Hz), 7.54–7.62 (m, 4H), 8.25 (d, 2H, *J* = 8.1 Hz). Not observed (brs, 2H, NH_2_). ^13^C-NMR (100 MHz DMSO-*d*_6_) δ: 14.4 (CH_3_), 24.4 (CH_2_), 25.5 (CH_2_), 26.1 (CH_2_), 31.1 (CH_2_), 33.6 (CH_2_), 52.0 (CH_3_), 105.1 (C), 114.4 (C), 115.5 (CH), 117.9 (C), 120.8 (CH), 125.4 (C), 126.6 (CH), 129.6 (CH), 130.0 (CH), 138.5 (C), 145.8 (C), 147.5 (C), 147.6 (C), 149.5 (C), 152.9 (C), 157.4 (C), 161.0 (C). *m/z*: 449 (M^+^,1), 434 (3), 236 (10). Anal. Calcd for C_28_H_27_N_5_O: C, 74.81; H, 6.05; N, 15.58; found: C, 74.84; H, 6.01; N, 15.62.

### 3.3. Antifungal Activity

#### 3.3.1. Microorganisms and Media

For the antifungal evaluation, standardized strains from the American Type Culture Collection (ATCC), Rockville, MD, USA, Reference Center in Mycology (CEREMIC, CCC, Rosario, Argentina) were used. Standardized strains: *C. albicans* ATCC 10231 and *C. neoformans* ATCC 32264; clinical isolates of *Candida* genus were provided by CCC. Voucher specimens of the isolated are presented in Table 5. Strains were grown on Sabouraud-chloramphenicol agar slants for 48 h at 30 °C, were maintained on slopes of Sabouraud-dextrose agar (SDA, Oxoid) and sub-cultured every 15 days to prevent pleomorphic transformations. Inocula were obtained according to reported procedures, [51] and adjusted to 1 × 10^3^–5 × 10^3^ cells with colony forming units (CFU)/mL.

#### 3.3.2. Fungal Growth Inhibition Percentage Determination

Yeasts broth microdilution technique M27-A3 of CLSI [51] was performed in 96-well microplates. For the assay, compound test wells (CTWs) were prepared with stock solutions of each compound in DMSO (maximum concentration ≤ 1%), diluted with RPMI-1640, to final concentrations of 250–3.9 μg∙mL^−1^. An inoculum suspension (100 μL) was added to each well (final volume in the well = 200 μL). A growth control well (GCW) (containing medium, inoculum, and the same amount of DMSO used in a CTW, but compound-free) and a sterility control well (SCW) (sample, medium, and sterile water instead of inoculum) were included for each fungus tested. Microtiter trays were incubated in a moist, dark chamber at 30 °C for 48 h for both yeasts. Microplates were read in a VERSA Max microplate reader (Molecular Devices, Sunnyvale, CA, USA). Amphotericin B was used as positive control. Tests were performed in triplicate. Reduction of growth for each compound concentration was calculated as follows: % of inhibition = 100 − (OD 405 CTW − OD 405 SCW)/(OD 405 GCW − OD 405 SCW). The means ± SD (standard deviations) were used for constructing the dose-response curves representing % inhibition *vs.* concentration of each compound. Dose-response curves were constructed with SigmaPlot 11.0 software.

#### 3.3.3. MIC_100_, MIC_80_ and MIC_50_ Determinations

Three endpoints were defined from the dose-response curves. Minimum Inhibitory concentration (MIC) resulting in total fungal growth inhibition was named MIC_100_, while MIC_80_ and MIC_50_ were defined as the minimum concentration that inhibits 80% or 50% of the fungal growth, respectively. 

### 3.4. Anticancer Activity

The human tumor cell lines of the cancer-screening panel were grown in RPMI 1640 medium containing 5% fetal bovine serum and 2 mM L-glutamine. For a typical screening experiment, cells are inoculated into 96 well microtiter plates. After cell inoculation, the microtiter plates were incubated at 37 °C, 5% CO_2_ and 95% air, and 100% relative humidity for 24 h prior to addition of tested compounds. After 24 h, two plates of each cell line were fixed *in situ* with TCA, to represent a measurement of the cell population for each cell line at the time of sample addition (T_z_). The samples were solubilized in dimethyl sulfoxide (DMSO) at 400-fold the desired final maximum test concentration and stored frozen prior to use. At the time of compounds addition, an aliquot of frozen concentrate was thawed and diluted to twice the desired final maximum test concentration with complete medium containing 50 µg∙mL^−1^ gentamicin. Additionally, four 10-fold or ½ log serial dilutions were made to provide a total of five drug concentrations plus control. Aliquots of 100 µL of these different sample dilutions were added to the appropriate microtiter wells already containing 100 µL of medium, resulting in the required final sample concentrations [58]. After the tested compounds were added, the plates were incubated for an additional 48 h at 37 °C, 5% CO_2_ and 95% air, and 100% relative humidity. For adherent cells, the assay was terminated by the addition of cold TCA. Cells were fixed *in situ* by the gentle addition of 50 µL of cold 50% (w/v) TCA (final concentration, 10% TCA) and incubated for 60 min at 4 °C. The supernatant was discarded, and plates were washed five times with tap water and air-dried. Sulforhodamine B (SRB) solution (100 µL) at 0.4% (w/v) in 1% acetic acid was added to each well, and plates were incubated for 10 min at room temperature. After staining, unbound dye was removed by washing five times with 1% acetic acid and the plates were air-dried. Bound stain was subsequently solubilized with 10 mM trizma base, and the absorbance was read on an automated plate reader at a wavelength of 515 nm. Using the seven absorbance measurements (time zero (T_z_), control growth in the absence of drug (C), and test growth in the presence of drug at the five concentration levels (T_i_)), the percentage growth was calculated at each of the drug concentrations levels. Percentage growth inhibition was calculated as: [(T_i_ − T_z_)/(C − T_z_)] × 100 for concentrations for which T_i_ > T_z_, and [(T_i_ − T_z_)/T_z_] × 100 for concentrations for which T_i_ < T_z_. Three dose response parameters were calculated for each compound. Growth inhibition of 50% (GI_50_) was calculated from [(T_i_ − T_z_)/(C − T_z_)] × 100 = 50, which is the drug concentration resulting in a 50% lower net protein increase in the treated cells (measured by SRB staining), as compared to the net protein increase seen in the control cells. The drug concentration resulting in total growth inhibition (TGI) was calculated from T_i_ = T_z_. The LC_50_ (concentration of drug resulting in a 50% reduction in the measured protein at the end of the drug treatment as compared to that at the beginning), indicating a net loss of cells following treatment, was calculated from [(T_i_ − T_z_)/T_z_] × 100 = −50. Values were calculated for each of these three parameters if the level of activity is reached; however, if the effect was not reached or was exceeded, the value for that parameter was expressed as greater or less than the maximum or minimum concentration tested [58,59,60,61].

## 4. Conclusions

In this article we described the microwave-assisted synthesis of novel pyrazolo[3,4-*g*][1,8]naphthyridin-5-amine **3** by the Friedländer condensation of *o*-aminonitrile **1** with cyclic ketones **2** using zinc chloride as a catalyst. The presented synthetic procedure is an environmentally friendly, simple, and high yielding method for the preparation of compounds **3**. The whole series of compounds **3** were tested against standardized strains of the clinically important fungi, *C. albicans* and *C. neoformans*, showing *C. albicans* to be more sensitive to the whole synthetic series than *C. neoformans*. Compounds **3a**, **3d** and **3g**, all possessing a *p*-tolyl substituent, but different fused rings, were the most active structures. They showed good antifungal activity against a second panel of clinical isolates of *albicans* and non-*albicans Candida* species and appear as good models for the development of new analogues with improved activity. The antitumor evaluation data revealed that compounds **3a** and **3e** exhibited remarkable activity with GI_50_ values in the range from 10−6 M against different cancer cell lines.

## Supplementary Materials

Supplementary materials can be accessed at: http://www.mdpi.com/1420-3049/20/05/8499/s1.
