# Supplementary material for: Microwave-Assisted Synthesis of Novel Pyrazolo[3,4-g][1,8]naphthyridin-5-amine with Potential Antifungal and Antitumor Activity
Source: Molecules. 2015 May 12;20(5):8499–520. doi: 10.3390/molecules20058499 (PMC6273193; doi:10.3390/molecules20058499)
Supplement: Supplementary file 1 [file molecules-20-08499-s001.pdf]

Supplementary Materials

Table S1. Percentages of inhibition of **3a–i** against *C. albicans* ATCC 10231 and *C. neoformans* ATCC 32264 within the range 250–3.9 µg/mL.

**Sub-SEries**	**Moiety**	**R**	**Comp**	**Concentrations in µg/mL**						
				250	125	62.5	31.2	15.6	7.8	3.9
	furane	CH_3	**3a**	100	100	88.81 ± 4.91	20.58 ± 0.01	6.00 ± 3.50	1.82 ± 0.08	0.36 ± 0.04
i.1	cyclohexyl	CH_3	**3d**	100	100	100	100	24.68 ± 7.61	5.11 ± 0.81	5.27 ± 1.86
	cycloheptyl	CH_3	**3g**	100	100	57.66 ± 0.44	21.38 ± 0.49	0	0	0
	furane	Cl	**3b**	87.25 ± 1.81	43.63 ± 0.34	9.08 ± 0.98	0	0	0	0
i.2	cyclohexyl	Cl	**3e**	100	56.19 ± 2.46	15.02 ± 1.59	7.55 ± 1.48	3.09 ± 2.19	0	0
	cycloheptyl	Cl	**3h**	100	100	33.95 ± 0.75	0	0	0	0
	furane	OCH_3	**3c**	100	35.58 ± 1.95	13.57 ± 0.99	8.30 ± 0.78	3.02 ± 1.11	2.17 ± 2.76	2.64 ± 0.50
i.3	cyclohexyl	OCH_3	**3f**	100	56.35 ± 0.21	20.58 ± 1.10	9.27 ± 0.32	0	0	0
	cycloheptyl	OCH_3	**3i**	100	50.55 ± 4.88	13.73 ± 0.54	8.51 ± 0.15	0.39 ± 1.37	0	0
C. neoformans ATCC 32264										
	furane	CH_3	**3a**	100	63.40 ± 1.76	49.49 ± 8.81	47.95 ± 0.82	23.34 ± 8.61	17.59 ± 2.03	0
i.1	cyclohexyl	CH_3	**3d**	100	55.03 ± 1.22	15.96 ± 8.99	11.97 ± 5.50	10.68 ± 4.56	7.04 ± 0.91	0
	cycloheptyl	CH_3	**3g**	100	100	37.43 ± 1.83	8.97 ± 1.09	5.07 ± 0.88	3.21 ± 0.43	0
	furane	Cl	**3b**	75.21 ± 1.70	25.65 ± 0.80	16.91 ± 1.33	19.99 ± 0.43	19.07 ± 0.52	17.09 ± 0.63	17.02 ± 0.75
i.2	cyclohexyl	Cl	**3e**	54.53 ± 5.52	35.49 ± 3.33	26.87 ± 5.81	23.10 ± 5.07	12.59 ± 11.02	7.44 ± 6.54	0
	cycloheptyl	Cl	**3h**	100	76.33 ± 1.46	20.58 ± 0.84	18.82 ± 0.23	10.02 ± 0.18	2.54 ± 3.07	1.00 ± 0.02
	furane	OCH_3	**3c**	80.26 ± 3.40	61.00 ± 3.06	52.06 ± 9.06	49.81 ± 0.39	44.52 ± 2.98	30.01 ± 15.74	28.30 ± 8.95
i.3	cyclohexyl	OCH_3	**3f**	100	84.34 ± 5.13	27.22 ± 9.16	25.77 ± 4.22	11.02 ± 1.44	7.29 ± 1.79	0
	cycloheptyl	OCH_3	**3i**	74.14 ± 2.16	51.92 ± 2.19	15.85 ± 1.51	10.51 ± 7.42	0	0	0
	Amphotericin B			100	100	100	100	100	100	100
